# Production of a potential multistrain probiotic in co-culture conditions using agro-industrial by-products-based medium for fish nutrition

**DOI:** 10.1186/s12896-023-00822-5

**Published:** 2023-12-15

**Authors:** Marcelo Fernando Valle-Vargas, Ruth Yolanda Ruiz-Pardo, Luisa Villamil-Díaz, María Ximena Quintanilla-Carvajal

**Affiliations:** Grupo de Investigación en Procesos Agroindustriales (GIPA), Doctorado en Biociencias, Facultad de Ingeniería, Universidad de La Sabana. Campus del Puente del Común, Autopista Norte de Bogotá. Chía, Km. 7, Cundinamarca, Colombia

**Keywords:** Whey, Molasses, Palm kernel cake, Co-culture, *L. lactis*, *Priestia*, Fermentation

## Abstract

**Background:**

Probiotics are viable microorganisms that when administered in adequate amounts confer health benefits to the host. In fish, probiotic administration has improved growth, and immunological parameters. For this reason, it is necessary production of probiotic bacteria, however, commercial culture mediums used for probiotic growth are expensive, so the design of a “low” cost culture medium is necessary. Therefore, this research aimed to produce a potential multistrain probiotic preparation composed of *L. lactis* A12 and *Priestia* species isolated from Nile tilapia (*Oreochromis niloticus*) gut using an agro-industrial by-products-based culture medium.

**Results:**

A Box-Behnken design with three factors (whey, molasses, and yeast extract concentration) was used. As the main results, a high concentration of three components enhanced the viability of *L. lactis* A12, however, viable cell counts of *Priestia* species were achieved at low molasses concentrations. The Optimal conditions were 1.00% w/v whey, 0.50% w/v molasses, and 1.50% w/v yeast extract. *L. lactis* A12 and *Priestia* species viable counts were 9.43 and 6.89 Log_10_ CFU/mL, respectively. *L. lactis* A12 concentration was higher (*p* < *0.05*) in the proposed medium compared to commercial broth.

**Conclusions:**

It was possible to produce *L.* *lactis* A12 and *Priestia* species in co-culture conditions. Whey and molasses were suitable components to produce the multistrain preparation. The cost of the proposed culture medium was 77.54% cheaper than the commercial medium. The proposed culture medium could be an alternative to commercial mediums for the production of this multistrain probiotic.

## Background

According to the Food and Agriculture Organization of the United Nations, the worldwide population will reach 9.2 billion people by 2050, this means that food production must increase by 50% [1]. With this perspective, aquaculture is a growing industry that could meet this demand for the increasing global population [2]. The high production of aquaculture species has caused the propagation of diseases that have been treated with antibiotics, however, the inadequate use of this substance has led to the occurrence of antimicrobial resistance microorganisms [3]. Because of this, researchers are studying environmentally friendly alternatives to antibiotics to treat diseases and improve growth parameters in aquaculture species [4]. These alternatives include biofloc-based system [5, 6], vaccines [7], medicinal plants [8, 9], algae [10, 11], prebiotics [12, 13], probiotics [14–16], etc. Among these, probiotics have been reported to improve gut health in fish and shellfish species [4].

Probiotics are viable microorganisms that when administered in adequate amounts confer health benefits to the host [17]. Probiotics represent a wide group of bacteria, mostly lactic acid bacteria (LAB), other gram-positive bacteria like *Bacillus* spp*.,* and yeast like *Saccharomyces cerevisiae* [18, 19]. These microorganisms have been used in animal feeding, including fish [20]. Probiotics as feed additives confer benefits to the host, such as improving weight gain, nutrient digestibility, immunomodulation, gut microbiota modulation, and resistance against pathogens [21]. Probiotics have been produced under laboratory, pilot, and industrial bioreactor conditions [22] to produce biomass (microorganisms) in large quantities and/or bioactive compounds [23]. Commercial culture mediums are used, because they contain carbohydrates, amino acids, and minerals, among other nutrients which microorganisms need to grow. However, these culture mediums are expensive, due to nitrogen sources such as peptones, beef extract, yeast extract, and others [24–26]. The culture medium represents 30 – 40% of the total cost of probiotic production [27]. For this reason, the use of agro-industrial by-products is an alternative that can be used as a culture medium component for probiotic production. These components included whey, molasses, palm kernel cake, cereal straw, soy flour, etc. Moreover, these components have been used in fish nutrition, so it is not necessary downstream processes like centrifugation and washing for cell recovery [28–30]. Probiotics can be produced in *monoculture* or *monostrain* (single bacteria) and *co-culture* or *multistrain* (two or more microorganisms). Production of probiotic bacteria in co-culture conditions has shown higher cell viability and antimicrobial activity than in monocultures [31], which could be related to additive o synergistic effects [32]. Some researchers have reported positive effects of co-culture production, in the case of Gutiérrez–Cortés et al. [33] who found that the co-culture of *Lactobacillus plantarum* and *Pediococcus pentosaceus* in a whey-based medium increased bacteriocin production expressed as arbitrary units (AU) by *P. pentosaceus* from 19,200 UA/mL (monoculture) to 52,000 AU/mL (co-culture). Ariana and Hamedi [34] evaluated the effect of a co-culture of *Lactococcus lactis* and *Yarrowia lipolytica* in a molasses-based medium on *L. lactis* growth and nisin production. The authors found that this co-culture increased nisin production and *L. lactis* growth by 50% and 49% higher than *L. lactis* in monoculture, respectively.

Some probiotic bacteria, such as LAB and *Bacillus* species, have attracted great interest in the scientific community for their ability to improve growth parameters and resistance against pathogens in fish species at a laboratory scale [20, 35]. Melo-Bolívar et al. [36] characterized the microbial community composition of a continuous-flow competitive exclusion culture (CFEC) from gut microbiomes of Nile tilapia (*Oreochromis niloticus*), from which some bacteria were isolated. Three isolates (*L. lactis* A12, *Priestia megaterium* M4, and *Priestia* sp. M10) from the CFEC showed tolerance to acid pH, bile salts, antibacterial activity against pathogens, such as *Streptococcus agalactiae* and *Aeromonas hydrophila*, etc. [37]. Most recently, these bacteria were used to develop a bacterial consortium using a mixture design to evaluate the effect of the initial inoculum proportion on the growth rate and antibacterial activity of cell-free supernatants against *Streptococcus agalactiae* and *Aeromonas hydrophila* [38]*.* These authors found that two combinations of the probiotic bacteria showed the highest growth rate and antibacterial activity against fish pathogens. One combination was composed by 61% v/v strain A12, 23% v/v strain M10, and 16% v/v strain M4. The other combination was 72% v/v strain M10 and 28% v/v strain M4. Finally, these authors used these probiotic bacteria in single and multistrain preparations in an in vivo experiment and found that both preparations improved growth performance, gut histology, gut microbiota, immune regulation, and infection resistance in Nile tilapia fingerlings [14]. However, these bacterial consortia were grown in BHI broth. For this reason, it is necessary as a first step to evaluate the production of these bacteria in co-culture conditions using agro-industrial by-products such as whey, sugarcane molasses, and PKC as components in a culture medium, which is the scope of this research.

The importance of this research lies in addressing the need for cost-effective production of probiotic bacteria under co-culture conditions, as commercial culture mediums used for probiotic growth are known to be expensive.

To the best of our knowledge, there are no reports in the literature on the production of the probiotic consortium proposed in this study using agro-industrial by-products. Therefore, this research aimed to produce a potential multistrain probiotic preparation using an agro-industrial by-product culture medium composed of whey, sugarcane molasses, and palm kernel cake. Additionally, an evaluation of probiotic characteristics under optimal conditions in a 1.7 L lab bioreactor scale was conducted.

## Materials and methods

### Ethical statement

The project followed the Colombian national government’s regulations. The Permit for accessing genetic resources was issued by the Colombian Ministry of Environment Number 117 (Otrosí4) on the 8th of May 2018 for five years.

### Microorganisms

*L. lactis* A12, *P. megaterium* M4, and *Priestia* sp*.* M10 were isolated from a competitive exclusion bacterial culture derived from the Nile tilapia (*O. niloticus*) gut microbiota [36]. Potential probiotic bacteria were identified using molecular techniques and sequenced the whole genome [37]. Bacteria were deposited under codes A12 (*L. lactis* A12), M4-MR4 (*Priestia megaterium* M4), and M10-MR10 (*Priestia* sp. M10) in the Chilean Collection of Microbial Genetic Resources (CChRGM) at the Instituto de Investigaciones Agropecuarias (INIA, Chillan, Chile). This institute is registered at the World Data Centre for Microorganisms (WDCM) with registration number 1067. These bacteria were stored in 1.5 mL Eppendorf tubes with BHI (Oxoid, UK) and 40% v/v glycerol at -20 °C in a bacterial suspension: BHI with a volume ratio of 1:1. Bacteria were activated on TSA (Tryptic Soy Agar, Sharlau, Spain) at 28 °C for 48 h. Then, a single colony was taken from the TSA, inoculated in BHI broth (Brain Heart Infusion, Oxoid, UK), and incubated overnight at 28 °C [38].

### Preparation of culture medium and fermentation conditions

Whey powder (Saputo, Colombia), sugarcane molasses (VitaAgro, Colombia), yeast extract (Oxoid, UK), and PKC (Hacienda La Cabaña, Colombia) were used as culture medium components. PKC was grounded and sieved through a 1.0 mm mesh and added to the mixture at 0.77% w/v. The approximate composition of culture medium components is presented in Table 1. The Components were mixed in different proportions according to the experimental design (see Sect. 2.3.) and added to distilled water for a final volume of 45 mL in a 250 mL shake flask. Di-sodium phosphate (Merck, Germany) was used as a buffering agent at 2.63% w/v. The final mixture was sterilized at 121 °C for 15 min. Then, the culture medium (45 mL) was inoculated with 5 mL of bacterial inoculum and placed in an orbital incubator shaker (Innova 42, New Brunswick Scientific, USA) at 75 RPM and 28 °C for 24 h. The bacterial inoculum was composed of 61% v/v *L. lactis* A12, 23% v/v *Priestia* sp. M10, and 16%v/v *P. megaterium* M4, as reported by Melo-Bolívar et al. [38]. The initial bacterial count of *L. lactis* A12 and *Priestia* species was 4.41 ± 0.13 and 4.37 ± 0.13 Log_10_ CFU/mL, respectively. Viable cell counts were performed at the end of the fermentation process by the plate count method in TSA at 28 °C for 24 h. The bacterial count was expressed as Log_10_ CFU/mL [39].

**Table 1:** Composition of culture medium components

Wet basis (% w/w)	Whey powder	Sugarcane molasses	PKC
Moisture	4.28	14.50	5.78
Ashes	6.50	6.23	5.32
Lipids	0.15	0.00	10.91
Carbohydrates	78.08	76.79	36.81
Protein	10.99	2.48	14.62
Crude fiber	0.00	0.00	24.76

### Experimental design

In a previous study, optimal conditions for producing probiotic bacteria in monoculture were achieved (data under submission). These conditions were whey (3.84% w/v), sugarcane molasses (7.39% w/v), PKC (0.77% w/v), and 75RPM. However, it was necessary to evaluate the viability of these bacteria under co-culture conditions. Preliminary experiments had to be carried out (data not shown) to established new concentration ranges for whey sugarcane molasses, and yeast extract were necessary for the co-culture of probiotic bacteria. For this purpose, a Box-Behnken design (BBD) was used to optimize the component concentration that maximizes the viability of *L. lactis* A12 and *Priestia* species (*P*. *megaterium* M4 and *Priestia* sp. M10). The agitation speed and PKC concentration were kept constant with values of 75 RPM and 0.77% w/v, respectively.

The culture medium design was optimized with a BBD, which was built using the statistical software Design Expert (Stat-Ease Inc., Minneapolis, MN, U.S.A) [39]. The design consisted of 15 runs, with three replicates at the central point (see Table 2). The medium components were considered as numerical factors: whey (1.00—3.84% w/v), sugarcane molasses (0.50 – 3.16% w/v), and yeast extract (1.50 – 3.50% w/v). The response variables were the viability of *L. lactis* A12 and *Priestia* species expressed as Log_10_ CFU/mL.

**Table 2:** Experimental matrix of Box Behnken design of viability of probiotic bacteria in co-culture

Run	Whey (% w/v)	Molasses (% w/v)	Yeast extract (% w/v)	*Priestia* species (Log_10_ CFU/mL)	*L. lactis* A12 (Log_10_ CFU/mL)
1	1.00	0.50	2.50	6.33 ± 0.02	9.25 ± 0.05
2	2.42	0.50	1.50	6.72 ± 0.07	9.26 ± 0.02
3	1.00	3.16	2.50	5.31 ± 0.15	9.71 ± 0.03
4	2.42	1.83	2.50	6.23 ± 0.07	9.66 ± 0.06
5	3.84	3.16	2.50	5.39 ± 0.00	9.52 ± 0.08
6	2.42	1.83	2.50	6.30 ± 0.07	9.56 ± 0.09
7	3.84	1.83	3.50	5.26 ± 0.09	9.87 ± 0.03
8	1.00	1.83	3.50	6.24 ± 0.04	9.87 ± 0.04
9	3.84	0.50	2.50	6.77 ± 0.11	9.68 ± 0.06
10	2.42	3.16	1.50	4.62 ± 0.06	9.79 ± 0.04
11	2.42	1.83	2.50	6.37 ± 0.06	9.68 ± 0.04
12	3.84	1.83	1.50	5.63 ± 0.03	9.85 ± 0.03
13	2.42	0.50	3.50	6.59 ± 0.05	9.63 ± 0.10
14	2.42	3.16	3.50	5.24 ± 0.09	9.70 ± 0.07
15	1.00	1.83	1.50	6.63 ± 0.03	9.56 ± 0.07

The optimal component concentration in the culture medium that maximized the viability of probiotic bacteria under co-culture conditions was achieved using the desirability function. The criterion of desirability is a general approach in which the value of each response variable is transformed into a measurement ranging from 0 to 1; values close to 1 represent maximization processes, whereas values close to 0 represent minimization processes [40, 41]. The validation of the response variables was performed at optimal conditions. Also, the model prediction was validated using two additional points. The error percentages of the predicted and experimental data were calculated. Validation runs were performed in triplicate.

Finally, the optimal conditions in co-culture were compared to those in BHI using a *t-test* at a 95% of the level of confidence. In addition, homogeneity of variance for the *t-test* was confirmed using a *F-test*.

### Production of probiotic bacteria in a lab-scale bioreactor and evaluation of probiotic characteristics

After optimal conditions were achieved in a 250 mL shake flask, the next step was to produce this potential multistrain probiotic in a 1.7 L bioreactor. For this reason, probiotic bacteria were produced in a bioreactor with a working volume of 1 L. Activation and inoculum preparation of bacteria was performed according to the methodology described in Sect. 2.1. Next, a 250 mL shake flank containing 90 mL of BHI broth (previously sterilized) was inoculated with 10 mL of inoculum (61% v/v *L. lactis* A12, 23% v/v, *Priestia* sp. M10, and 16% v/v *P. megaterium* M4). The inoculated BHI broth was placed in an orbital incubator shaker (Innova 42, New Brunswick Scientific, USA) at 28 °C and 100 RPM for 7 h. In the meantime, 900 mL of culture medium was prepared according to the optimal conditions described in Sect. 2.3. Distilled water was then added up to a volume of 900 mL in a 1.7 L bioreactor. The final mixture was sterilized at 121 °C for 15 min. After that, the culture medium contained in the bioreactor was inoculated with the 7-h bacteria grown in BHI broth. The bioreactor conditions were set as follows: agitation speed (100 RPM), temperature (28 °C), and incubation time (17 h). Finally, after the process was completed, samples of the final culture medium with the probiotic bacteria were taken to evaluate the final cell concentration (Log_10_ CFU/mL), tolerance to acid pH (bacterial reduction), tolerance to bile salt (bacterial reduction), and antibacterial activity against *Streptococcus agalactiae* (inhibition zone, mm) [37].

#### Viability of probiotic bacteria in co-culture

A bacterial sample (1 mL) obtained after 17 h of incubation in the bioreactor was added to 9 mL of saline solution (0.89% w/v). Ten-fold serial dilutions were made. Viable cell counts were determined using the plate count method on TSA at 28 °C for 24 h. The bacterial counts for *L. lactis* A12 and *Priestia* species were expressed as Log_10_ CFU/mL [38].

#### Tolerance to acidic pH and bile salts

A bacterial sample (1 mL) obtained after 17 h of incubation in the bioreactor was mixed with 9 mL of simulated gastric solution (acid or bile salts) contained in a 50 mL falcon tube. Then, Falcon tubes were agitated at 50 RPM and 28 °C for 2 h [37]. Then, 1 mL of each falcon sample was added to 9 mL of phosphate buffer solution. Ten-fold serial dilutions were made. The final viable cell count was determined using the plate count method on TSA at 28 °C for 24 h. As a control, 1 mL of the initial sample was mixed with 9 mL of saline solution (0.89% w/v). The tolerance of bacteria to simulated gastric conditions was expressed as the bacterial reduction after 2 h of exposure [37].

The acid-simulated solution was prepared by adding HCl solution to BHI broth until a pH of 3.00 was reached. Bile gastric solution was prepared by adjusting BHI broth to pH 7.00 and adding a bile salt combination (Sigma Aldrich, U.S.A) to a concentration of 0.30% w/v. Both solutions were sterilized at 121 °C for 15 min [39].

#### Antibacterial activity against Streptococcus agalactiae

*S. agalactiae* was stored in 1.5 mL Eppendorf tubes with BHI (Oxoid, UK) (40% v/v glycerol) at -20 °C in a bacteria suspension: BHI volume ratio of 1:1. Bacteria were activated, on TSA (Tryptic Soy Agar, Sharlau, Spain) at 28 °C for 48 h. Then, a single colony was taken from the TSA and inoculated in 8 mL of BHI broth (Brain Heart Infusion, Oxoid, UK), and incubated overnight at 28 °C. Next, the overnight inoculum was adjusted to a cell density of approximately 6.00 Log_10_ CFU/mL, and it was used to inoculate TSA plates by streaking evenly across the agar surface using a sterile cotton swab [36]. After 20 min, three sterile paper filter disks with a diameter of 7 mm were put on the TSA surface and each was inoculated with 30 µL of the final culture medium sample. Then, TSA plates were incubated at 28 °C for 48 h. Finally, antibacterial activity was expressed as the inhibition zone (mm) around the paper disk where the pathogen bacteria have not grown enough to be visible [42].

## Results

### Model fitting of response variables

Table 2 presented the experimental values of the viability of *L. lactis* A12 and *Priestia* species. To analyze the data, quadratic and linear models were fitted to the viability of *L. lactis* A12 and *Priestia* species, respectively. Table 3 shows that models were selected based on the lowest *p-value*, indicating a significant effect. The models for *L. lactis* A12 and *Priestia* species explained 96.04% and 72.18% of the total variability in the experiments, respectively. Another important statistical parameter is the Adequate precision, which measures the signal-to-noise ratio. The Adeq precision values for *L. lactis* A12 and *Priestia* species models were 12.80 and 8.86, respectively, indicating that both models can be used to navigate the experiment design space effectively.

**Table 3:** ANOVA and statistical parameters of the viability of *L. lactis* A12 and *Priestia* species

	*p- value*	
	*L. lactis* A12	*Priestia* species
Model	0.0053	0.0022
A—Whey	0.0318	0.2227
B—Sugarcane molasses	0.0041	0.0003
C—Yeast extract	0.0193	0.8155
AB	0.0045	-
AC	0.0711	-
BC	0.0152	-
A^2^	0.1936	-
B^2^	0.0075	-
C^2^	0.0249	-
Fitting parameters		
R^2^	0.9604	0.7218
R^2^_adj_	0.8890	0.6460
Adeq Precision	12.8082	8.8604

The viability of *L. lactis* A12 was found to be significantly affected by the concentration of whey (A), sugarcane molasses (B), and yeast extract (C), with *p-values* less than 0.05. Additionally, the linear interactions (AB and AC) and quadratic terms (B^2^ and C^2^) also had a significant impact on the response variable. In the case of the *Priestia* species model, only the concentration of sugarcane molasses was found to have a significant effect on the viability of these species during the co-culture experiment, with a *p-value* less than 0.05. The coded equations for *L. lactis* A12 and *Priestia* species are presented as Eqs. 1 and 2, respectively. These equations are useful to determine the relative impact of factors by comparing factor coefficients or coefficient estimates. Coefficient estimates indicate the expected change in the response variable per unit change in factor value when all other factors remain constant. The coded equation allows us to predict the response for a given level of each factor, with high levels of factors represented as + 1 and low levels as -1. In the case of *L. lactis* A12, the viability is positively influenced by A, B, C, and C^2^, while the interaction and quadratic terms have a negative impact. On the other hand, for *Priestia* species, only factor B has a significant and negative impact on viability.

1 $$\begin{gathered} \begin{array}{*{20}{l}} {L.{\text{ }}lactis\left( {Lo{g_{10}}CFU/mL} \right)}&= \end{array}9.63+0.0662 \times \left[ A \right]+0.1125 \times \left[ B \right]+0.0762 \times \left[ C \right] \hfill \\ - 0.1550 \times \left[ {AB} \right] - 0.0725 \times \left[ {AC} \right] - 0.1150 \times \left[ {BC} \right]+0.00496 \times \left[ {{A^2}} \right] - 0.1429 \times \left[ {{B^2}} \right]+0.1046 \times \left[ {{C^2}} \right] \hfill \\ \end{gathered}$$

2 $$Priestia~species{\text{ }}\left( {Lo{g_{10}}CFU/mL} \right)~=5.998 - 0.1825 \times \left[ A \right] - 0.7313 \times \left[ B \right] - 0.0337 \times \left[ C \right]$$

### Effect of independent variables on the viability of *L. lactis *A12 and *Priestia* species

**Fig. 1:**
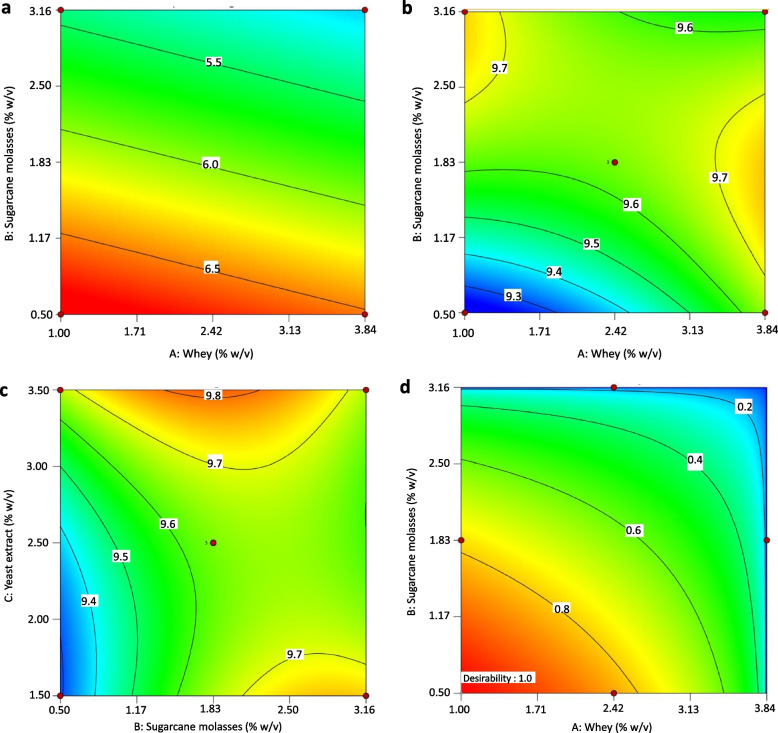
Contour plots for *Priestia* species (**a**), *L. lactis* A12 (**b** and **c**), and desirability function (**d**)

Table 2 presents the results of the Box-Behnken design conducted for the production of probiotic bacteria in co-culture conditions. The viability of *Priestia* species varied from 4.62 to 6.77 Log_10_ CFU/mL. As shown in Fig. 1a, it was observed that a high concentration of sugarcane molasses led to a low concentration of viable cells in the final culture medium. On the other hand, *L. lactis* A12 viability values ranged from 9.25 to 9.87 Log_10_ CFU/mL. Figure 1b shows a whey-sugarcane molasses concentration interaction. It is evident that high concentrations of whey and molasses concentration improve the viability values. Figure 1c shows the interaction between sugarcane molasses and yeast extract concentration. In this figure, it can be seen the same trend was observed in whey-molasses interaction, high concentration of molasses and yeast extract resulted in high viability of *L. lactis* A12.

### Optimization and validation of optimal conditions

The desirability function was used to optimize the concentration of culture medium components for maximizing the viability of *L. lactis* A12 and *Priestia* species. The desirability value increased when whey concentration and sugarcane molasses decreased, while maintaining a yeast extract concentration of 1.50% w/v. The optimal conditions for achieving a desirability value of 1.00 were: 1.00% w/v whey, 0.50% w/v sugarcane molasses, and 1.50% w/v yeast extract. Desirability values higher than 0.7 indicate a good optimization of experimental data. Table 4 shows the predicted and experimental values of *L. lactis* A12 and *Priestia* species under optimal conditions and two points for model validation. Experimental errors for *L. lactis* A12 and *Priestia* species were 4.31 and -0.43%, respectively, for optimal conditions. For model validation purpose, two randomly points were selected: point 1 (2.94% w/v whey, 1.83% w/v sugarcane molasses, and 3.50% w/v yeast extract) and point 2 (1.68% w/v whey, 1.83% w/v sugarcane molasses, and 3.50% w/v yeast extract). Experimental errors for *L. lactis* A12 in point 1 and 2 were -0.33 and 0.29%, respectively. For *Priestia* species, these values were -2.91 and 3.92%, respectively. With experimental errors values lower than 10%, indicates that the desirability function was a useful statistical tool for the optimization of culture medium components. In co-culture conditions, the viability of probiotic bacteria was compared to that obtained using BHI broth. Notably, *L. lactis* A12 exhibited significantly higher viability (*p* < *0.05*) in the proposed culture medium compared to BHI broth. However, the viability of *Priestia* species in our medium was lower (*p* < *0.05*) compared to BHI broth.

**Table 4:** Validation of optimal conditions and model points in co-culture

	Predicted value (Log_10_ CFU/mL)		Observed value (Log_10_ CFU/mL)	
	*L. lactis* A12	*Priestia* species	*L. lactis* A12	*Priestia* species
Optimal	9.04	6.92	9.43 ± 0.02^b^	6.89 ± 0.10^a^
BHI	N. A	N. A	9.25 ± 0.10^a^	7.55 ± 0.17^b^
Model points				
Point 1	9.82	5.87	9.79 ± 0.03	5.70 ± 0.00
Point 2	9.68	5.44	9.71 ± 0.08	5.65 ± 0.00

N.A: This condition was carried out for comparison purposes. Different superscripted letters ^(a-b) ^within the same column indicate significant difference (*p* < *0.05*)

The estimated cost of the proposed culture medium based on the price of its components was $5.17 USD per liter, as indicated in Table 5. In comparison, BHI broth had a cost of $23.04 USD per liter.

**Table 5:** Estimation of total cost of culture medium

Component	Concentration (g/L)	Price ($ USD/kg)	Total price ($ USD)	Cost contribution (%)
Whey powder	10.00	3.22	0.032	0.62
Sugarcane molasses	5.00	0.66	0.003	0.06
PKC	7.70	0.13	0.001	0.02
Yeast extract	15.00	189.34	2.84	54.93
Na_2_HPO_4_	26.30	87.30	2.30	44.48
Cost per liter of medium	-	-	5.17	
BHI (cost per liter)	-	-	23.04	

Cost contribution (%) = (component cost / cost per liter o medium) × 100

### Production of probiotic bacteria in a lab-scale bioreactor and evaluation of probiotic characteristics

Table 6 presents the probiotic characteristics of the co-culture conditions that were evaluated using a 1.7 L lab bioreactor with a working volume of 1 L. The evaluation was conducted over a 17-h incubation period.

**Table 6:** Probiotic characteristics of co-culture in a lab bioreactor

	*L. lactis* A12	*Priestia* species
Final viability (Log_10_ CFU/mL)	9.47 ± 0.06	6.72 ± 0.01
Tolerance to acid pH (bacterial reduction)	2.31 ± 0.21	0.00 ± 0.00
Tolerance to bile salt (bacterial reduction)	1.32 ± 0.12	0.00 ± 0.00
Antibacterial activity (mm)	12.0 ± 1.0	

## Discussion

Probiotics confer health benefits to the host, including improvements in growth parameters, nutrient absorption, immune response, among others [43]. Normally, commercial growth media are used for probiotic production, but these media are highly expensive and must be centrifuged and washed for their inclusion in animal feed [44]. To reduce production cost, it is necessary to develop alternative culture medium. In this regard, agro-industrial by-products could be used as components for culture media production of probiotics at a lower cost [39, 45–48]. The components proposed in this research are by-products from dairy (whey), sugar (molasses), and palm oil (PKC) industries. These components are sources of carbon (glucose, sucrose, fructose, lactose) and nitrogen (proteins) source [47, 49, 50].

Although *Priestia* species of this work could metabolize several monosaccharides (ribose, mannose, fructose, glucose, galactose) and disaccharides (sucrose and lactose), as well as biosynthesis of several amino acids and vitamins [37]. However, higher concentrations of molasses decrease *Priestia* species cell count. This behavior could be related to the presence of inhibitory substances such as heavy metals [47], aluminum, sulfites, thermal sugar degradation compounds [46, 51, 52] and /or high concentrations of sugars that could cause osmotic stress [53].

The viability of *L. lactis* A12 was enhanced when high concentrations of whey, sugarcane molasses, and yeast extract were used. Melo-Bolívar et al. [37] evaluated the genome of *L. lactis* A12 and found that this bacteria has genes associated with metabolism of various monosaccharides (ribose, mannose, fructose, glucose, galactose) and disaccharides (sucrose and lactose), as well as biosynthesis of several amino acids and vitamins. According to the literature, some of these carbon sources are present in whey [33, 45], sugarcane molasses [34, 52], and PKC [54]. These components have been included in culture mediums for the production of probiotic microorganisms such as lactic acid bacteria and *Bacillus* species.

Also, yeast extract had been used as a supplement in culture medium for the production of various bacterial species including *Lactobacillus plantarum* [46, 48, 55], *Lactobacillus fermentum* [39], *Lactococcus lactis* [56], *B. subtilis* [54], and *B. licheniformis* [52]. Yeast extract is a source of amino acids, peptides, nucleic acid derivates, and minerals. Additionally, yeast extract is a source of B-complex vitamins that stimulate bacterial growth [25].

While the use of BHI medium resulted in better viability values for *Priestia* species, it should be noted that commercial mediums contain nitrogen sources such as peptones, and beef extract, among others, which contribute to the high cost of bacteria growth media [25]. Although using the proposed medium resulted in lower *Priestia* species than BHI, *L. lactis* A12 cell count was higher in the proposed culture medium. This suggest that the optimal mixture could be suitable for biomass production of these bacteria. In contrast, BHI medium contains nitrogen sources such as peptone protease (10 g/L), brain infusion (12.5 g/L), and beef heart infusion (5 g/L) solid. These components contribute to the higher cost of the bacteria growth media [25]. Also, BHI broth contains glucose (2.0 g/L) as sole carbon source and sodium phosphate as buffering agent (2.5 g/L). The proposed culture medium consists of whey (10 g/L), sugarcane molasses (5 g/L), PKC (7.7 g/L), yeast extract (15 g/L), and sodium phosphate (26.3 g/L). It is important to highlight that whey, sugarcane molasses, and PKC are used by probiotic bacteria as carbon and nitrogen source, and more important are the most cost-effective components of the culture medium. Also, yeast extract is used as nitrogen source, which concentration in culture medium is lower than sources in BHI (27.5 g/L). Despite the proposed culture medium used ten times more sodium phosphate (26.30 g/L) than BHI (2.5 g/L) and, both yeast extract and sodium phosphate account for 99.41% of the total cost of culture medium, our culture medium cost 77.54% less than BHI. Therefore, the proposed culture medium is a “low cost” alternative to commercial medium to produce probiotics intended for fish feed supplementation.

The final cell count in a bioreactor for *L. lactis* A12 (9.47 Log_10_ CFU/mL) was close to those reported by Costas-Malvido et al. [49, 56] in re-alkalinized fed-batch whey-based medium supplemented with K_2_HPO4 and MRS broth nutrients, respectively. These studies were carried out in a 13L bioreactor with a working volume of 10L. Other studies have reported viability values ranging from 8.00 to 10.00 Log_10_ CFU/mL for the production of *L. fermentum* [39], *L. paracasei* [47], *P. pentosaceus* [33], where whey or molasses were used as components for growth media.

Additionally, Norizan et al. [54] reported maximum cell counts for biomass production of *Bacillus subtilis* using PCK as a medium component in both flask and bioreactor resulting in cell counts of 9.47 and 5.45 Log_10_ CFU/mL, respectively. It is important to highlight that these fermentations lasted 72 h. In all studies described above, by-products were identified as a “low-cost” alternative for probiotic production.

The reduction of probiotic bacteria under acidic and bile salts conditions was consistent with the findings reported by Aragón-Rojas et al. [39]. They investigated the effect of whey, yeast extract, pH, and agitation conditions on viable cell count, lag phase, reduction in acidic and bile salts conditions of *Lactobacillus fermentum* K73 produced in a 1L-lab bioreactor with a working volume of 800 mL. The study conducted by Melo-Bolívar et al. [37] assessed the probiotic potential of bacteria in mono-culture conditions and found that *Priestia* species resisted acidic and bile salts conditions after 2 h. However, *L. lactis* A12 did not survive acidic conditions after 2 h. It is important to highlight that *L. lactis* A12 grown in co-culture in the proposed medium did survive pH and bile salts conditions after 2 h. On the other hand, *Priestia* species did not survive in acidic and bile salt environments.

Furthermore, the culture medium containing potential multistrain probiotics exhibited antibacterial activity against *S. agalactiae*. Melo-Bolívar et al. [38] reported that cell-free supernatant obtained from a bacterial consortium consisting of *L. lactis* A12, *Priestia megaterium* M4, and *Priestia* sp. M10 grown in BHI showed antibacterial activity against *S. agalactiae* and *Aeromonas hydrophila*. It was reported that in the genome of *L. lactis* A12, *Priestia megaterium* M4, and *Priestia* sp. M10 were found genes related to the production of bacteriocins, namely Lactococcin, Paeninodin, and Bacteriocin uviB, respectively [37].

## Conclusions

The co-culture of *L. lactis* A12 and *Priestia* species was successfully achieved using an agro-industrial by-product medium comprising whey, sugarcane molasses, and palm kernel cake. This medium, which served as a “low-cost” source of nutrients, supported the growth of the potential probiotic bacteria consortium in a lab-scale bioreactor under optimal conditions. The resulting culture exhibited probiotic characteristics, including viability, tolerance to an acidic environment, tolerance to bile salts, and antibacterial activity against *Streptococcus agalactiae*. These findings suggest that the proposed culture medium has the potential to be used for producing a multistrain probiotic composed of *L. lactis* A12, *Priestia megaterium* M4, and *Priestia* sp. M10, offering an alternative to commercial mediums. The estimated cost of the culture medium, based on the price of its components, was 77.54% cheaper than BHI broth. This cost reduction was achieved by using low-cost components such as whey, sugarcane molasses, and palm kernel cake as carbon and nitrogen sources, which collectively represented only 0.71% of the total cost of the culture medium. In contrast, yeast extract and sodium phosphate were identified as the most expensive components, suggesting the need for further research to optimize their inclusion in the culture medium. Additionally, the utilization of agro-industrial by-products for bacteria production offers the advantage of avoiding downstream processes like centrifugation and washing. Moreover, it enables the generation of a double-purpose culture medium that promotes the growth of probiotic bacteria (including potential bioactive compounds produced by the bacteria) and facilitates their stabilization through encapsulation.

## Data Availability

The datasets used and/or analyzed during the current study are available from the corresponding author on reasonable request.
